# Supplementary material for: The interactome of intact mitochondria by cross-linking mass spectrometry provides evidence for coexisting respiratory supercomplexes
Source: Mol Cell Proteomics. 2017 Dec 8;17(2):216–32. doi: 10.1074/mcp.RA117.000470 (PMC5795388; doi:10.1074/mcp.RA117.000470)
Supplement: Supplemental Data [file supp_RA117.000470_133922_0_supp_23978_fzffwf.zip › spectra_annotation/mito_spectra_annotation/161-1-3-1-9-1.pdf]

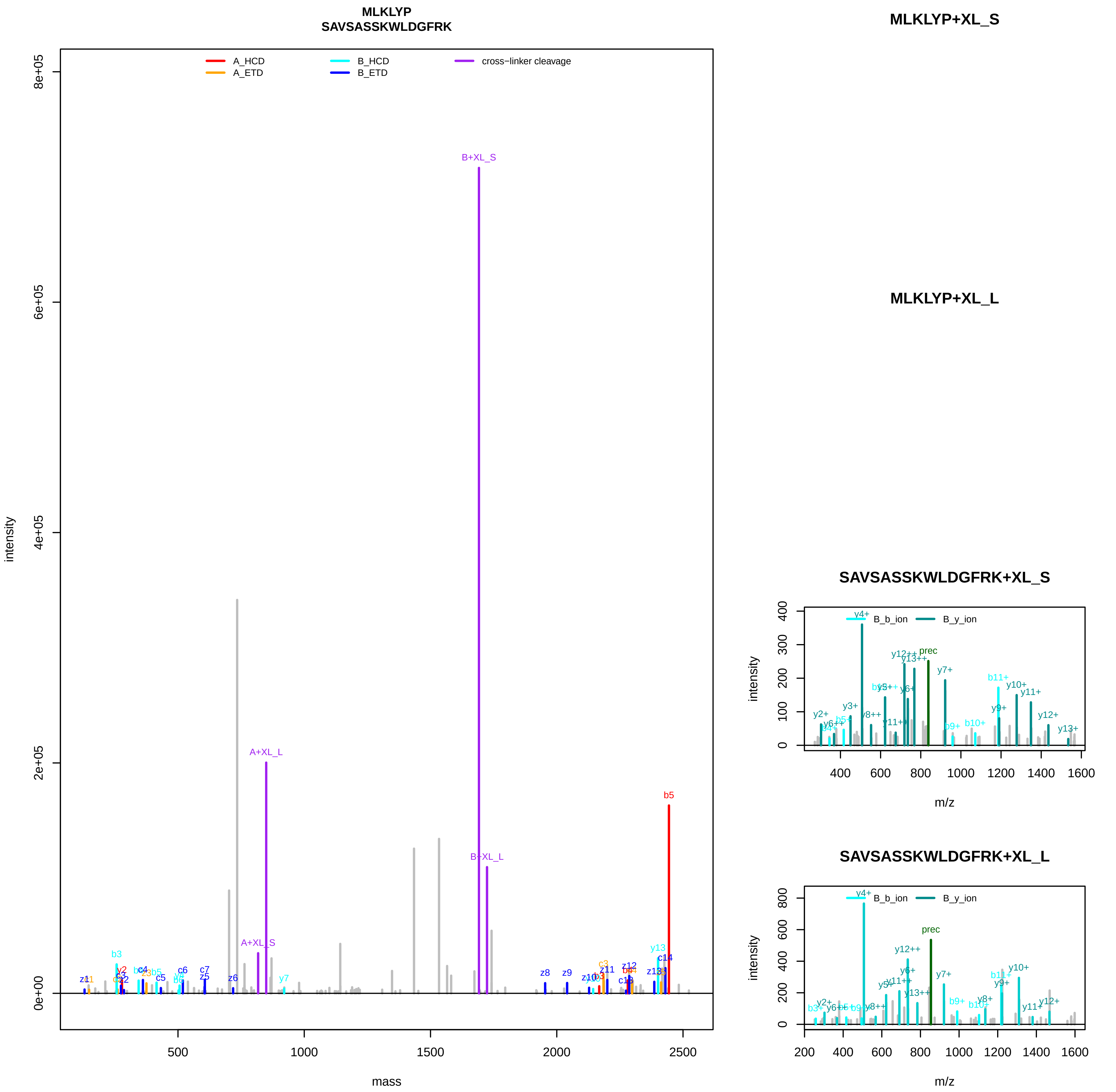

MLKLYP
SAVSASSKWLDGFRK
A_HCD
A_ETD
B_HCD
B_ETD
cross−linker cleavage
B+XL_S
A+XL_L
B+XL_L
A+XL_S
b5
y13
c14
z13
z12
c13
z11
z10
z9
z8
z6
c7
z5
c6
c5
c4
b3
y7
intensity
mass
0e+00
2e+05
4e+05
6e+05
8e+05
500
1000
1500
2000
2500
MLKLYP+XL_S
MLKLYP+XL_L
SAVSASSKWLDGFRK+XL_S
B_b_ion
B_y_ion
prec
y4+
y12++
y13++
y7+
b11+
y10+
y11+
y12+
y13+
y3+
y2+
y9+
y6+
b9+
b10+
intensity
m/z
0
100
200
300
400
400
600
800
1000
1200
1400
1600
SAVSASSKWLDGFRK+XL_L
B_b_ion
B_y_ion
prec
y4+
y12++
y7+
y10+
b11+
y9+
y13++
y12+
y11+
b9+
b10+
y8+
y2+
b3+
intensity
m/z
0
200
400
600
800
200
400
600
800
1000
1200
1400
1600
